# An Early Sensitive Period Induces Long-Lasting Plasticity in the Honeybee Nervous System

**DOI:** 10.3389/fnbeh.2018.00011

**Published:** 2018-02-01

**Authors:** Juan P. Grosso, Jesica A. Barneto, Rodrigo A. Velarde, Eduardo A. Pagano, Jorge A. Zavala, Walter M. Farina

**Affiliations:** ^1^Laboratorio de Insectos Sociales, Departamento de Biodiversidad y Biología Experimental, Facultad de Ciencias Exactas y Naturales, Universidad de Buenos Aires, Buenos Aires, Argentina; ^2^Instituto de Fisiología, Biología Molecular y Neurociencias (IFIBYNE), CONICET, Universidad de Buenos Aires, Buenos Aires, Argentina; ^3^Cátedra de Bioquímica, Facultad de Agronomía, Universidad de Buenos Aires, Buenos Aires, Argentina; ^4^Instituto de Investigaciones en Biociencias Agrícolas y Ambientales (INBA), CONICET, Universidad de Buenos Aires, Buenos Aires, Argentina

**Keywords:** plasticity, sensitive period, behavior, neurobiology, social insect

## Abstract

The effect of early experiences on the brain during a sensitive period exerts a long-lasting influence on the mature individual. Despite behavioral and neural plasticity caused by early experiences having been reported in the honeybee *Apis mellifera*, the presence of a sensitive period in which associative experiences lead to pronounced modifications in the adult nervous system is still unclear. Laboratory-reared bees were fed with scented food within specific temporal windows and were assessed for memory retention, in the regulation of gene expression related to the synaptic formation and in the olfactory perception of their antennae at 17 days of age. Bees were able to retain a food-odor association acquired 5–8 days after emergence, but not before, and showed better retention than those exposed to an odor at 9–12 days. In the brain, the odor-rewarded experiences that occurred at 5–8 days of age boosted the expression levels of the cell adhesion proteins neurexin 1 (*Nrx1*) and neuroligin 2 (*Nlg2*) involved in synaptic strength. At the antennae, the experiences increased the electrical response to a novel odor but not to the one experienced. Therefore, a sensitive period that induces long-lasting behavioral, functional and structural changes is found in adult honeybees.

## Introduction

In highly social insects, division of labor is based mainly on age polyethism, in which individuals carry out different sets of behaviors according to their age (Wilson, 1971). Age-related polyethism plays a crucial role in task allocation in the eusocial honeybee *Apis mellifera* (Michener, 1974). Newly emerged adult workers mostly clean the comb cells and nurse brood inside the hive while middle-aged bees process and store food until they start foraging tasks outside the nest on the third week after emergence (Rösch, 1925; Lindauer, 1952; Seeley, 1982). This age-related polyethism makes honeybees suitable models to analyze the effects of particular environmental stimuli or cognitive capacities experienced during young adulthood on later behavior, and their consequences at physiological and structural levels (Fahrbach and Robinson, 1996).

Forager bees can learn floral odors while visiting profitable food sources. Learning of odor cues can lead bees to memorize and store these associations in different neural substrates of the brain (Giurfa and Sandoz, 2012; Menzel, 2012; Meyer and Galizia, 2012), guiding foragers toward the learned stimuli in subsequent foraging trips (Dukas, 2008). However, not only this worker sub-caste can gain access to this information. Young adult hive bees not directly involved in foraging tasks can receive the collected nectar by direct food transfers via mouth-to-mouth interactions, acquiring information about the nourishment (Grüter et al., 2006; Farina et al., 2007; Martinez and Farina, 2008). Therefore, individuals that acquired information earlier in life could retrieve it later, if memories are long-lasting enough, within a novel behavioral context, such as while searching for food in the field.

Some prior reports suggested a more plastic behavior in young adult bees (Arenas and Farina, 2008; Behrends and Scheiner, 2009) than first thought (Ray and Ferneyhough, 1997; Morgan et al., 1998; Ichikawa and Sasaki, 2003). A previous study (Arenas and Farina, 2008), showed that olfactory memories established during a specific period a few days after emergence can be retrieved at the third week of adult life (17 days old). The same study showed that the strength of memory retention is age-dependent. So, when associations occurred between 5 days and 8 days of adult age, individuals achieved better olfactory retention than if the same odor-reward associations occurred before (1–4 days) or even after (9–12 days) this period (Arenas and Farina, 2008; Arenas et al., 2009a). This age-dependent effect of early learning was observed only in bees reared under control laboratory conditions, not in individuals reared inside the hive (Arenas and Farina, 2008). This emphasizes the complex interplay between the ontogeny of the olfactory pathway, the age of acquisition and the rearing environment (Arenas et al., 2013).

Moreover, olfactory memories established later than the period of 5–8 days of age are better retrieved if honeybees have been exposed to an odor-rewarded experience within the same period (Arenas et al., 2009a), but not if bees have been exposed to the odorant as a volatile in the rearing environment (i.e., not associated with sucrose solution). This result suggests a relationship between the development of proper associative learning and memory retention capabilities at older ages and the exposure to a learning event at younger ages. Thus, early odor memories established at 5–8 days of age might modify structure and function relations before the olfactory system is finally mature (Masson and Arnold, 1987; Masson et al., 1993; Winnington et al., 1996; Farris et al., 2001). A conditioned odor acquired via scented food 5–8 days after emergence evokes enhanced activity of the first olfactory center of the insect brain, the antennal lobe (AL), modifying its spatiotemporal response patterns (Arenas et al., 2009b). This reorganization translates into structural changes, since increases in volume for the subunits of neuropils of the AL, the glomeruli, appear to be specific to the learned odor (Arenas et al., 2012). Some reports show that associative odor learning in young adult bees is not identical to odor learning in older bees (Faber et al., 1999; Sandoz et al., 2003). When mature adult workers acquire long-term memories and variations in glomerular volume, they do not show changes in glomerular activity patterns (Hourcade et al., 2009). In contrast, when bees acquire long-term memories at 5–8 days of age, they present a positive correlation between glomerular activity and volume change at foraging ages (Arenas et al., 2012). These pieces of evidence suggest that a putative sensitive period within the interval of 5–8 days of age might be taking place. Previous studies focused on analyzing neurobiological outputs of odor-rewarded experiences occurring within this specific interval (Arenas et al., 2009b, 2012). However, structural and physiological approaches that consider different pre-foraging age periods involved in information acquisition are needed to define this period properly.

The repeated activation of specific synapses by experience has been postulated as one of the mechanisms participating in a sensitive period (Knudsen, 2004). This involves the insertion of cell adhesion molecules (CAMs), which structurally consolidate the synapse, making it invulnerable to subsequent elimination (Benson et al., 2000). Among CAMs, there are highly conserved proteins located in the synaptic membranes of neurons that form a binding pair: the presynaptic *neurexins* (*Nrxs*) and their postsynaptic binding partners, the *neuroligins* (*Nlg*s). Together, they form a trans-synaptic bond that serves synapse formation (Craig and Kang, 2007) and, as such, they participate in the learning processes. For example, *Neurexin-1 (Nrx1)* is required for synapse formation and associative learning in *Drosophila* larvae (Zeng et al., 2007). New evidence indicates that the regulation of synaptic connectivity in adult honeybees changes after a sensory experience occurs (Biswas et al., 2010; Reinhard and Claudianos, 2012). In the honeybee brain, expression levels of these molecules are always present throughout development and they are significantly up-regulated during adulthood (Dean and Dresbach, 2006; Biswas et al., 2008). Interestingly, expression of both types of molecules was found to be up-regulated in the brain of foraging aged bees after having been successfully conditioned to an odor (Biswas et al., 2010). This opens up the possibility to detect memory traces using the expression levels of this molecules in the adult honeybee brain.

Early stimulation may alter other pathways by which experience may persist, including changes in the peripheral nervous system. Changes in the response of the insects’ antennae due to olfactory deprivation and experiences with odors have been reported. When bees are deprived of olfactory stimulation since emergence, adults show a decreased antennal response to different novel odors honeybees: (Masson and Arnold, 1984); *Drosophila*: (Devaud et al., 2003). The first wave of studies showed that electroantennogram (EAG) recordings increased after odor learning (de Jong and Pham-Delègue, 1991; Wadhams et al., 1994). However, other authors did not detect an effect of the conditioned odors (Bhagavan and Smith, 1997; Sandoz et al., 2001). In recent years, a reduction in EAG response was reported for honeybees pre-exposed to odor-rewarded experiences during adulthood and also during preimaginal stages (Claudianos et al., 2014; Ramírez et al., 2016). Such a reduction seems to correlate with a down-regulation of olfactory receptors linked to conditioned odors, which implies a selectively lower responsiveness in the EAG to experienced odors while enabling the animal to remain receptive to new floral odors (Claudianos et al., 2014).

With this in mind, honeybees reared under laboratory conditions were fed with scented food within specific temporal windows and were assessed at 17 days of age for memory retention, regulation of gene expression related to the binding synaptic proteins and olfactory perception of the antennae. To test for memory retention, the proboscis extension response (PER) paradigm was used to perform single odor tests at the PER setup (Takeda, 1961). Assessment of synaptic formation has been addressed by measuring gene expression of the CAMs found in the honeybee brain (Dean and Dresbach, 2006; Biswas et al., 2008). Olfactory perception at the sensory periphery has been approached by testing the effect of early odor-rewarded stimulation on the electrophysiological response of whole antennae of adult bees.

## Materials and Methods

### Treatments and Rearing Conditions

European honeybees *Apis mellifera* from the experimental apiary located at the Faculty of Exact and Natural Sciences of the University of Buenos Aires, Argentina (34° 32’ S, 58° 26’ W) were used for all experiments, during the summer seasons of 2014–2016. Brood frames containing capped brood were transferred from the hives to an incubator (constant darkness, RH 55%, 32°C). Every day, newly emerged bees (i.e., between 0 h and 24 h old) were placed into wooden cages (10 × 10 × 10 cm) in groups of 60–120 bees. Cages were placed in a second incubator (RH 55%, 31°C and darkness). Bees were allowed to emerge from each frame during four consecutive days. One cage was set up per day corresponding, in random order, to each treatment group. Caged bees were provided with *ad libitum* 50% w/w sucrose solution during the entire period by using 10 ml plastic test tubes with a hole in the bottom as feeders. Pollen paste was offered during the first 10 days as a protein source (Williams et al., 2013). Every 2 days, the sucrose solution was replaced to prevent fungi and bacteria proliferation.

**Figure 1:**
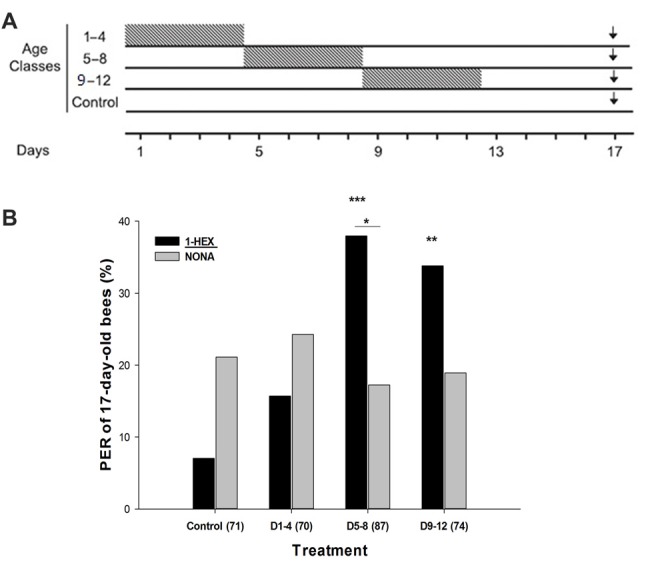
**(A)** Schematic schedule of the experimental series along the adult lifespan of the honeybee. Caged bees reared throughout their adult lifespan in incubators were fed 1-HEXANOL (1-HEX) scented sugar solution during four consecutive days (gray boxes), while the rest of the experimental period were fed unscented sugar solution. At 17 days of age (black arrow) behavioral and neurobiological variables were tested. This determines the following treatments: control group, bees were fed unscented solution during the whole experimental period of 17 days; D1–4 group, bees were fed 1-HEX sucrose solution only between the first and fourth days; D5–8 group, bees were fed 1-HEX sucrose solution only between the fifth and eighth days; D9–12 group, bees were fed 1-HEX sucrose solution only between the ninth and twelfth days.** (B)** Proboscis extension response (PER) to the early experienced odor (1-HEX) and to a novel odor (NONA) in bees of 17-days of age that underwent a controlled odor-rewarded experience at specific adult age periods. Significant differences in PER values compared to control with odor (1-HEX) are labeled with **Pr(>|z|) < 0.01 and ***Pr(>|z|) < 0.005 and within the same treatment is labeled with *Pr(>|z|) < 0.05 (Generalized Linear Mixed Model, GLMM test). The number of observations (bees from that treatment) is shown between brackets.

Treated bees were fed with sucrose solution scented with 1-HEX at 50 μl/l for the EAG recordings and gene expression measurements and at 100 μl/l for the behavioral experiment (concentration was increased due to lack of PER with the 50 μl/l). 1-HEX was diluted in 50% sucrose solution and offered to the caged bees during four consecutive days in different adult periods (Arenas et al., 2008): in the D1–4 treatment bees received the scented sucrose solution during the first 4 days of the adult lifespan, and then the sucrose solution was unscented for the remaining experimental period (Figure 1A); in the D5–8 treatment bees received 1-HEX scented sucrose solution only during the period of 5–8 days; in the D9–12 treatment caged bees received scented sucrose solution only during the period of 9–12 days. Except for the antennal recording experiment, the sucrose solution consumed during this scented food period was estimated. The Control bees were fed unscented sucrose solution during the whole 17 day period. At 17 days of age, individual bees were collected for testing (Figure 1A).

### Testing Behavioral Responses in PER Setup

Cold anesthetized bees were harnessed and left in an incubator (RH 55%, 31°C and darkness) in order to let them recover and settle in their new harnesses. Time of recovery varied greatly between individuals. We found that, in order to carry out the test with a maximum number of alert bees, we had to wait 1.5 h. However, the first bees to recover were too tired or hungry at this point so, to enable them to continue, we found that feeding them after 1 h in the incubator worked best. We offered a few drops of sucrose solution (50% w/w). Only bees awake and willing were fed with the sucrose solution at this point in time. After 30 more minutes, bees were taken out of the incubator and their capacity to perform PER in response to sucrose was assessed without feeding them. Bees unable to perform PER were discarded. For the testing phase, filter papers (20 mm × 4 mm size) embedded with 4 μl of pure odor were put individually into a syringe and an air flow controlled by a valve was directed through the syringe during 6 s, after second 16 of a 39 s long protocol. Odors were presented in a random order. Every time a bee extended its proboscis, we noted 1, if not, 0. Statistical analyses were performed using a Generalized Linear Mixed Model (GLMM, Baayen et al., 2008) with binomial error distribution and logit link function. The final model fit consisted of treatment and odor stimulation as fixed factors, their interaction, and bee as a random factor. This minimum model was found using backward stepwise fitting, random factor cage Pr(>Chisq) = 0.9775 was discarded and the interaction of fixed factors was kept Pr(>Chisq) = 1.959e-05. GLMM models were fitted in R 3.2.1(R Development Core Team, 2011) using the function lmer of the R-package lme4 (Bates and Maechler, 2010).

### qRT-PCR Measurements for Pre and Postsynaptic Proteins Expression Levels

Bees were cold anesthetized (3 min at −20°C) and brains were dissected. Bees were decapitated one at a time, and each head was fixed in melted wax for dissection. Each brain was dissected under a Leica MZ8 stereomicroscope. Microscissors and forceps were used for dissections. The brains of ten bees from the same cage (i.e., same treatment) were pooled in a single cryovial and conserved in liquid N_2_ until RNA extraction. Total RNA was extracted from each set of pooled brains with a TRIZOL extraction protocol (Invitrogen Life Technologies, MA, USA). Total RNA was treated with DNase, prior to reverse-transcription (RT) reactions. cDNA was synthesized (Revertaid RT, Thermo Fisher Scientific) with an input of 1 μg of total RNA. qPCR reactions were performed in triplicate to assess the expression level of *Nlgs 2–5* and *Nrx1*, using previously validated primers (Table 1) as in Biswas et al. (2008). In order to be able to compare relative abundance levels between genes, we used the relative quantification with standard curve method (Hellemans et al., 2007). Dilutions for the standard curves were prepared (1:3, 1:9, 1:27 and 1:81 seriated dilution of a cDNA stock made out from a pool of all samples) in order to generate a standard curve that was run in each plate and for each target gene alongside the unknown samples. The Applied Biosystems 7500 software was used to calculate the relative abundance values (Biosystems, 2004). Melting curve analysis was used to ensure amplification specificity. Rpl8 was used as the endogenous control (Collins et al., 2004).

**Table 1:** Primers models, amplified size product and annealing temperature for the constitutive gene (*Rpl8*) as well as for *neurexin 1 (Nrx1)* and *neuroligins 2–5 (Nlg 2, Nlg 3, Nlg 4* and *Nlg 5*).

Primer	Sequence (5′to 3′)	Product size (MW)	Annealing (°C)
*Rpl8*	F: CACACGGTGGTGGTAATCAT	114 pb	56
	R: CTCGGATTCTTCCTGTACGA
*Nlg2*	F: GGTGTTCCTCCTCGTGCTCAA	68 pb	59
	R: ACGAGTTCCTGTCCCTCTGGTA
*Nlg3*	F: CATAGAGCTCAAGTCGAAACTGAA	124 pb	56
	R: GAGAAGATGATGCGATCTAGGAA
*Nlg4*	F: CTTCCTGATTCTCGTCTGTCTGA	71 pb	56
	R: GTGGATTCAGCTTGCTCTTGA
*Nlg5*	F: GGTTGTATTCTGTTGGTGCTCAATA	67 pb	55
	R: TGTCTCGATCCCTCTGATAGTAAA
*Nrx1*	F: TCGAGTTCAAGACCGAGCA	81 pb	57
	R: GCTTCGCCTCGAAGAAGTC

*These models were made up using the Primer 3 software. Their thermodynamics properties were evaluated with the Invitrogen Vector NTI Advance™ 10 software*.

MANOVA analysis was used to assess significant differences between treatment groups across all genes and then individual ANOVAs were run for each gene across treatments to determine which genes were responsible for the differences found in the MANOVA, using the Infostat software.

### Electroantennogram (EAG) Recordings

The left antenna from each cold anesthetized bee was cut at the base of the scape and at the flagellum tip, to ensure that the electric circuit was closed. The electric conductive gel (SPECTRA 360 GEL) was used at the tip and base, which were placed one at each arm of a holder (Syntech) connected to a custom-made amplifier connected to a PC. Odor presentation lasted 1 s, interstimulus time was 1 min 13 s. Odor presentation or stimulation was random at increasing concentrations. Pico Protest, a custom-made software (by Máximo Lopez Medus) was used in order to control stimulus delivery and record electric peaks. Each antenna received all three dilutions of the two odors. For statistical analysis, LMM was performed using treatment and odor concentration as fixed factors, including their interaction (that was found significant Pr(>Chisq) 2.052e-06***) and antenna as a random factor. LMM models were fitted in R 3.2.1 (R Development Core Team, 2011) using the function glm of the R-package MASS (Ripley et al., 2013).

## Results

### Effect of Early Odor-Rewarded Experience on Later Behavioral Response

Honeybees extend their proboscises as an innate response to antennal and tarsal stimulation with sugared reward (Frings, 1944). Because this reflex response can be conditioned to an odor while they are harnessed (Takeda, 1961), we used the PER paradigm to perform single odor tests on 17-day-old bees that had undergone an odor-rewarded experience within a specific age period of 4 days (Figure 1A). During the PER test, we compared the bee responses to the odor offered during the specific period while they were reared in laboratory cages (1-Hexanol, 1-HEX) against a novel odor (Nonanal, NONA). As scented food presented during young adulthood improves the retrieval and acquisition of the odors experienced later in the bee life (Arenas and Farina, 2008) we predicted that prior presentation of 1-HEX scented food would elicit a high response level to the known odor. We reasoned that every time a bee was fed with 1-HEX scented sucrose solution in the laboratory cage during the 4 day period, a similar process to classical conditioning was taking place. We chose 1-HEX and NONA because they share a similar structure, both contain linear carbon chains with one functional group but are not easily confused by honeybees due to their difference in the perceptual space (Galizia and Kimmerle, 2004; Guerrieri et al., 2005).

The known odor 1-HEX elicited significantly more PERs than NONA for individuals from the D5–8 treatment group (Pr(>|z|) = 0.02186; Figure 1B). 1-HEX elicited higher PER levels for individuals from treatments D5–8 (Pr(>|z|) = 0.00274) and D9–12 (Pr(>|z|) = 0.00857), than in the control group. Differences between Control and D1–4 treatment were not found (Pr(>|z|) = 0.80247).

Additionally, during the scented food period, caged bees from the older pre-foraging age intervals consumed larger amounts of sucrose solution (D1–4, 50.8 ± 0.3 μl/bee, *N* = 5; D5–8, 75.7 ± 0.1, *N* = 4; D9–12, 119.1 ± 0.01, *N* = 3; *N* is the number of rearing cages measured).

### Effect of Early Odor-Rewarded Experience on Gene Expression in the Mature Bee Brain

**Figure 2:**
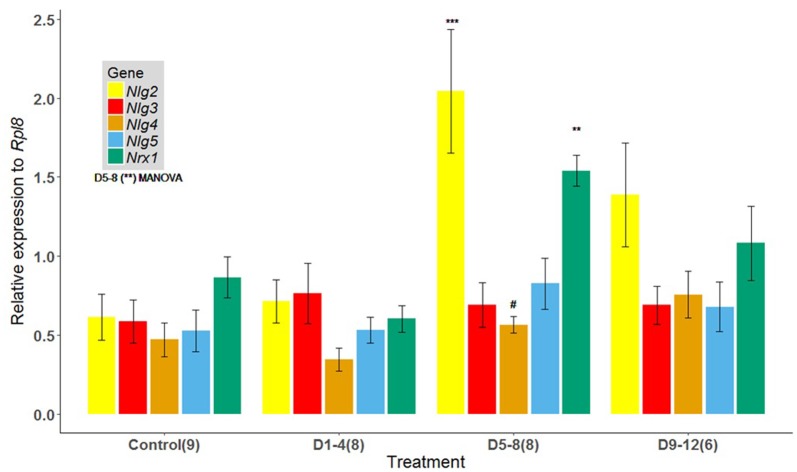
Expression of *Nlgs* and* Nrx1* in adult brains of bees of 17 days of age that underwent an early odor-rewarded experience during specific adult age periods. Expression of *Nlg2–5* and *Nrx1* in adult brain tissues between honeybees reared in cages fed 1-HEX-scented sucrose solution at 1–4, 5–8 or 9–12 days of age. The control group was fed unscented sucrose solution throughout the experimental period. Honeybee *Nlg2–5* and *Nrx1* expression were assessed by quantitative reverse transcription (RT) PCR amplification. The data are presented as fold change normalized to the endogenous reference gene *Rpl8*: mean values (± standard error).* Nlg2*, *neuroligin 2; Nlg3, neuroligin 3; Nlg4, neuroligin 4; Nlg5, neuroligin 5; Nrx 1, neurexin 1*. The number of brain pools is shown in brackets. Asterisks for D5–8 show significant differences between treatments (MANOVA *p* = 0.002, Pillai). Symbols on bars show differences from individual ANOVAs for *Nrx1* (*p* = 0.0003), *Nlg2* (*p* = 0.0018) and *Nlg4* (*p* = 0.055): ***P < 0.001, **P < 0.005, ^#^*P* < 0.10.

Evaluation of brains of 17-day-old bees showed significant differences in the levels of expression between treatments for the genes analyzed (*p* = 0.002, Pillai; Figure 2). Relative mRNA levels for *Nrx1* (*p* = 0.0003) and* Nlg2* (*p* = 0.0018) were significantly elevated for bees exposed to the known odor 1-HEX during the D5–8 interval, compared to bees exposed at time intervals D1–4, D9–12 and the control group (Figure 2). There were no significant differences in the levels of expression between treatment groups for *Nlg3* (*p* = 0.87) and* Nlg5* (*p* = 0.34), but it is worth noting a tendency for *Nlg4* even though it is not significant (*p* = 0.0555).

Moreover, bees consumed different volumes of 1-HEX-scented sucrose solution during the different scented food periods. The lowest consumption was achieved at the youngest age interval (D1–4, 27.9 ± 0.05 μl/bee, *N* = 3; D5–8, 75.4 ± 0.4, *N* = 4; D9–12, 84.4.1 ± 0.2, *N* = 4; N is the number of rearing cages measured).

### Effect of Early Odor-Rewarded Experience on Later Antennal Recording

This experiment was aimed to test the effect of early odor-rewarded stimulation on the electrophysiological response at the sensory periphery. EAG responses to the stimulus of three serial dilutions (1/100, 1/10 and 1/1) of the known odor, 1-HEX, and of a novel odor, NONA, were measured (same odor delivery system as in the PER setup, see “Materials and Methods” section).

**Figure 3:**
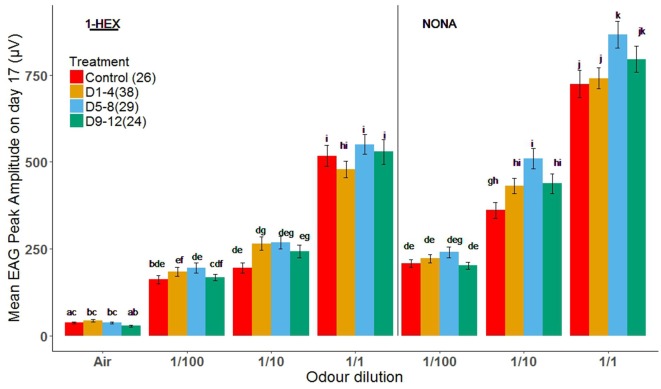
Electrical recordings of antennae from bees of 17 days of age that underwent a controlled odor-rewarded experience at different adult age periods. Mean values (± standard error) of electrical recordings of antennae of bees with (orange, light blue and green bars) and without (red bars) odor-rewarded experiences. Treated bees were fed 1-HEX-scented food at 1–4, 5–8 or 9–12 days of age and control bees fed unscented food. Three different concentrations of 1-HEX and NONA were tested. Letters indicate statistical differences in a Tukey of a linear mixed model (different letters indicate at least *P* < 0.05). The number of recordings per treatment is shown between brackets.

As expected, there was a concentration-dependent increase in response to both odors for 17-day-old bees in all treatment groups (Figure 3). In general, there were no significant differences in the response to all concentrations between treatment groups for 1-HEX. The same was true for NONA at the lowest dilution, 1/100. However, we identified a significant increase in the response of bees treated during D5–8 to the highest concentrations of NONA (1/10 and 1/1). EAG responses changed for D5–8 treated bees, compared to the control group, for the most concentrated samples of NONA (1/10 and 1/1; Tukey *p* = 0.0166 and 0.0311; Figure 3 respectively).

## Discussion

It is still unclear whether a limited and specific period in which sensory or associative experiences lead to pronounced modifications in the nervous system of the honeybee exists, even though the effect of experience on its brain is particularly strong during the first days of adulthood (Masson and Arnold, 1984, 1987; Arenas et al., 2009a, 2012). Our results show that odor-rewarded experiences that occur between the ages of 5–8 days after adult emergence have an impact on gene expression levels, odor detection at the sensory periphery and behavior in foraging aged honeybees. Regarding the expression levels of *Nrx* and* Nlgs*, individuals that experienced scented food in the D5–8 period had greater expression levels of *Nrx1* and *Nlg2*, two anchor protein partners that play a role in synaptic formation. At the sensory periphery, the known odor 1-HEX did not change across treatments physiological responses. However, the antennae showed higher EAG amplitudes for a novel odor (NONA) in individuals previously exposed to scented food within the D5–8 period. Furthermore, behavior did change due to early odor-rewarded experiences. PER levels towards the known odor were the highest for the same period compared with the control.

In the present study, we focused on the type of plasticity that involves synapse consolidation by CAMs. Repeated activation of these circuits by sensory stimuli during a sensitive period produces the insertion of CAMs, which structurally consolidate the synapse making it invulnerable to subsequent degradation (Benson et al., 2000; Knudsen, 2004). Accordingly, we found increased expression levels of *Nrx1* and* Nlg2* due to exposure to scented food during the D5–8 period. It is already known that *Nrx1* is the sole presynaptic binding protein partner of *Nlgs* in honeybees (Biswas et al., 2008). Its increased expression levels have been linked to odor-reward memory formation and rearing environment in honeybees (Biswas et al., 2010), as well as to the regulation of sleep, synaptic plasticity and associative learning in *Drosophila* (Zeng et al., 2007; Larkin et al., 2015).

Notably, we also found an increase in expression levels for postsynaptic binding protein partners *Nlg2* and* Nlg4* (a significant increase for *Nlg2* and a tendency for *Nlg4*). Biswas et al. (2008) reported that *Nlg2* shows the greatest change throughout honeybees’ lifespan (from preimaginal stages to foraging ages), with a 140-fold increase in expression between early development and the adult stage. The second greatest change throughout| development was reported in the same study for *Nlg4*, with a 70-fold increase in expression. This could explain why, in our study, *Nlg2* is the most sensitive of the Nlgs molecules within the D5–8 time frame. On the other hand, the highest expression levels for *Nlgs* after associative experiences in honeybees were found for *Nlg1* and* Nlg3* (Biswas et al., 2010). However, it is worth noting that differences in procedures exist between that study and the present one. While Biswas et al. (2010) measured mRNAs 48 h after training and used only forager bees, we focused on the effect of learning during young adulthood and tested it when bees are ready to start foraging duties. Moreover, we did not measure *Nlg1* expression as they did in that study. Thus, a pair of pre- and postsynaptic partner proteins overexpressed after early experiences is an effective mechanism for synaptic plasticity and one of the requirements for a sensitive period is met.

At the behavioral level, improved memory retention was found for bees from the D5–8 group and also for those from the D9–12 group. A better retention level for early ages has been reported previously, although with differences in the length of the exposed period depending on odorant identity (Arenas and Farina, 2008; Arenas et al., 2009a, 2012). For instance, when Linalool and Phenylacetaldehyde were used as food odors, the maximal retention was found only for the D5–8 group (Arenas and Farina, 2008) but if 1-HEX and 1-NON were used as food odors, the differences between D5–8 and D9–12 were weaker (Arenas et al., 2012). Despite of this, the use of 1-HEX in this study is based on the marked structural changes reported in the AL for the stimulation with this odorant during the D5–8 interval (Arenas et al., 2009b) and also on the high behavioral response found in adults after precocious experiences (Ramírez et al., 2016). In this sense, a sensitive period for neural and behavioral response development to learned odors found in rats shows that the preference for odors paired with reinforcing tactile stimulation was better at 19 days of age when the conditioning took place within the period of 1–8 post-natal days (PNDs), but this response decreased if the exposure period was reduced or delayed (1–4 and 8–14 PNDs; Woo and Leon, 1987).

The differences found in gene expression and behavior do not seem to correlate with scented sucrose consumption. While D9–12 is the period when the caged bees most consumed scented food, the response levels (gene expression) were lower than for D5–8 and D5–8 bees show they can behaviorally distinguish between 1-HEX and NONA while D9–12 could not. Although D1–4 was the period with least scented sucrose consumption, Arenas and Farina (2008) demonstrated that D1–4 bees can learn in the classical PER conditioning paradigm, so they are perfectly capable of learning the food odor association.

Because sensitive periods occur at specific levels of neural circuits, to avoid apparent contradictions one must analyze them within the circuit in which they happen to occur. In this study, antennae were analyzed on their own with the EAG in search of periphery specific changes in the electric response of the circuit. Treatments did not differ from the control group for the known odor and presented a dose-dependent response to increasing 1-HEX concentrations. Nonetheless, the amplitude of the peaks of bees from the D5–8 group increased for the novel odor, NONA, compared to the control group. Our EAG data does not replicate the study by Claudianos et al. (2014), although some differences in protocol exist. Meanwhile we studied long-term memory comprising several days, they analyzed it only 48 h after the first learning assay took place. Our results coincide with what is generally expected for odor receptors, although a decrease in the olfactory receptors specific to the odor learned 48 h before has been found (Claudianos et al., 2014). Once an odorant becomes familiar due to associative learning, it can be detected with a reduced number of olfactory receptors. Correspondingly, to allow honeybees to respond adaptively to their ever-changing scent environment, it could be advantageous to have other olfactory receptors comparatively upregulated (Menzel and Muller, 1996; Felsenberg et al., 2012). It seems that, over time, EAG amplitudes for the learned odor return to the values that naive bees have, but for D5–8 bees the responses increase for the novel odor in the long term (several days after learning took place). This effect that we see in the periphery may be due to feedback from the AL (Zwaka et al., 2016) or even higher centers; this connection remains to be inquired.

Masson et al. (1993) laid the foundations for identifying a sensitive period in honeybees when they established that between 3 days prior to emergence to 6 days after emergence the olfactory system is exposed to profound changes, although they did not look into learning and memory processes (Masson and Arnold, 1984, 1987; Gascuel and Masson, 1987, 1991). Taking into account the present results, it seems clear that at the end of this period (4–6 days after adult emergence) and for a window of 3–4 days, the anatomical, physiological and behavioral data point to a sensitive period. This period does not involve an irreversible imprinting-like process onto adult behavior because it is subject to extinction (Arenas et al., 2009b). It is also absent when bees are reared under the natural conditions of the hive (Arenas and Farina, 2008). Our study supports the hypothesis of a sensory period for the honeybee olfactory system from day 5 to day 8 after adult emergence, evidenced by behavioral variables, synaptic plasticity and responses at the sensory periphery.

How late in the development a sensitive period begins depends on the hierarchy of the circuitry that is experiencing it, because the reliable input information must proceed from the first sections of the circuitry that need to be already mature and functioning. Here, we investigate processes that depend on associative learning and memory for which the integration of information from different sensory modalities is necessary and usually occurs in the higher brain centers (Menzel, 2012). The behavioral studies performed need the whole circuitry working and the gene expression level ones used whole brain sample tissues, so it is difficult to pinpoint where the sensitive period is acting. Plus, the tests were carried out several days after the sensitive period had finished so we captured its sequels. Considering these issues, it is reasonable that the sensitive period for the honeybee olfactory system does not occur immediately after adult emergence. It remains to accurately study wherein the circuitry this is happening and why it is only noticeable when bees develop in cages, but not under the natural social context of the hive. In this sense, a natural rearing context implies a complex interplay between early experiences, age when they occurred and environment (Arenas and Farina, 2008; Arenas et al., 2013). The absence of prior odor experiences during the first 8 days of adult life in bees reared under laboratory conditions might explain the lower levels found for older bees in the responses studied (D9–12; see Arenas et al., 2009a).

In other model animals such as rats, it has been found that deprivation of social stimuli from PNDs 30 to 114 that correspond to adolescence and beyond, increases sucrose intake (Brenes et al., 2008) and enhances synaptic plasticity in a subcortical area which is critically involved in reward-based learning (Whitaker et al., 2013). Taking into account that social isolation affects the reward system and its plasticity, we can speculate that a similar process is in place in the honeybee, despite evolutionary divergence.

## Author Contributions

JPG, JAZ and WMF: conceived and designed the experiments; wrote the article. JPG, JAB and RAV: performed the experiments; analyzed the data. EAP, JAZ and WMF: contributed reagents/materials/analysis tools.

## Conflict of Interest Statement

The authors declare that the research was conducted in the absence of any commercial or financial relationships that could be construed as a potential conflict of interest.
